# Thermal Properties of Binary Filler Hybrid Composite with Graphene Oxide and Pyrolyzed Silicon-Coated Boron Nitride

**DOI:** 10.3390/polym12112553

**Published:** 2020-10-30

**Authors:** Jaehyun Wie, Jooheon Kim

**Affiliations:** School of Chemical Engineering and Materials Science, Chung-Ang University, Seoul 156-756, Korea; grwie23@naver.com

**Keywords:** polymer composite, thermal conductivity, surface modification

## Abstract

To improve the thermal conductivity of a composite material, the filler dispersion and the interfacial adhesion between the filler and the matrix are important factors. A number of methods for satisfying these criteria are presented herein. Thus, graphene oxide (GO) is incorporated to enhance the dispersion state of surface-modified boron nitride (BN) by increasing the viscosity of the epoxy matrix and by providing steric hindrance. Meanwhile, polysilazane (PSZ) coating and thermolysis were used to enhance the wettability by providing structural similarity between the coating material and the epoxy matrix. Due to these strategies, the thermal conductivity was improved by 253% compared to that of the neat epoxy at a filler fraction of 40 wt %.

## 1. Introduction

Since the efficient dissipation of accumulated heat is an important factor for the credibility and durability of electronic devices, thermal management has been an issue of great interest for many decades [1,2]. In this respect, the dielectric properties and excellent processability of polymers make these a popular choice for use as thermal interface materials (TIMs) [3]. However, the practical applicability of polymers is limited by their generally very low thermal conductivities (~0.2 W·m^−1^·k^−1^). To address this problem, various kinds of thermally conductive fillers have been used, including carbon-based, ceramic, and metal fillers. Boron nitride (BN) is generally used as a ceramic filler to improve the thermal conductivity and provide electrical insulation [4,5].

To improve the thermal conductivity, the filler dispersion and the interfacial adhesion in the composite are important issues [6,7]. Fillers tend to aggregate due to Van der Waals forces, thus leading to uneven dispersion in the composite [8]. This, in turn, affects the efficiency of the connecting networks for electrical and thermal transfer in the composite [9]. Meanwhile, structural incompatibility between rigid inorganic thermally conductive fillers such as BN and the polymer matrix often results in poor interfacial adhesion [10]. This causes phonon scattering and hinders phonon transfer by creating thermal boundary resistance. To address this problem, many researchers have tried various surface modifications for improving the affinity between the filler and the matrix [11,12,13].

Silicon-based materials such as polysilazane (PSZ) are used to coat substrates in order to provide chemical resistance and high thermal stability [14]. In addition, thermolysis is used to convert the PSZ into a ceramic [15]. The resulting structure has been shown to vary depending on the temperature of thermolysis, with the primary chain being converted from Si–N to Si–O at 1000 °C [16].

In the present study, several methods are used to address the various above-mentioned problems. The incorporation of graphene oxide (GO) is found to improve the filler dispersion due to the increased viscosity of the matrix and the steric hindrance caused by the GO. In addition, PSZ coating and thermolysis are used to cover the surface of the BN with siloxane groups and, thus, enhance the interfacial adhesion between the BN and the epoxy-terminated dimethylsiloxane (ETDS) matrix. Due to the heat transfer route provided by the enhanced dispersion and interfacial adhesion between the filler and the matrix, the obtained GO/P-BN/epoxy composite exhibited an improved thermal conductivity of 0.4517W/m^−1^·K^−1^, which is a 253% enhancement over that of the neat epoxy. Hence, this facile strategy was successfully used to improve the thermal conductivity of the polymer composite.

Fillers used in this paper were named as an abbreviated form such as R-BN (raw BN) and P-BN (polysilazane coated BN).

## 2. Experimental

### 2.1. Materials

Natural universal grade graphite flakes (~200 mesh, 99.9995% (metals basis)), and concentrated hydrogen peroxide (H_2_O_2_, 27% *w/w* aq.) were purchased from Alfa Aesar (St. Louise, MO, USA). Sulfuric acid (H_2_SO_4_, 95% aq.), phosphoric acid (H_3_PO_4_, 80% aq.), hydrochloric acid (25%, 20% aq.) were obtained from DaeJung Chemicals Co., Seoul, Korea. Potassium permanganate (KMnO_4_, 99%) and 4-4′-diaminodiphenylmethane (97%) were purchased from Sigma Aldrich (Seoul, Korea). Polysilazane (KiON HTT 1800) was supplied by Clariant GmbH (Muttenz, Switzer land). Epoxy resin (KF105; ETDS) was obtained from Shin-Etsu Silicon (Seoul, Korea). Boron nitride was obtained from LG Innotek (Seoul, Korea).

### 2.2. Preparation of Graphene Oxide (GO)

The GO was prepared from graphite flakes via an improved Hummers’ method [17]. Graphite (3 g) was first added to a 9:1 mixture of H_2_SO_4_/H_3_PO_4_ (400 mL) and the mixture was stirred for 4 h in an ice bath to produce a homogeneous dispersion. The KMnO_4_ (18 g) was then slowly introduced into the mixture and the reaction was stirred for 12 h at 323 K. After reaction, the mixture was cooled to room temperature (RT) and transferred to a solution of 30% H_2_O_2_ (5 mL) and 400 mL frozen de-ionized (DI) water. Finally, the mixture was centrifuged with HCl (400 mL) and purified with DI water. The resulting suspension was poured onto a glass slide and dried at 323 K for 72 h to yield the GO as a solid.

### 2.3. Surface-Modification of Boron Nitride (BN)

The BN (10g) and PSZ (1g) were stirred in acetone for 6 h at room temperature. The mixture was then washed twice with acetone before placing the precipitate in a convection oven at 323 K overnight. Finally, the dried powder was heated to 433 K for 4 h for the crosslinking reaction to occur, which was followed by pyrolysis at 1273 K for 2 h under an argon atmosphere. 

### 2.4. Fabrication of the GO/P-BN/Epoxy Composite

The GO was dispersed in ethanol using an ultrasonic bath for 1 h. Meanwhile, the ETDS and 4-4′-diaminodiphenylmethane curing agent (weight ratio of 5:1) were stirred on a hotplate at 403 K for 1h, followed by addition of the P-BN. The solution was maintained at this temperature under stirring until all traces of the solvent were removed; then, the obtained mixture was poured onto a Teflon plate and transferred to a vacuum oven at 353 K for 30 min to remove any air bubbles. The doctor blade technique was then used to control the uniformity of the film thickness. Finally, the composite film was cured in a furnace at 453 K for 1 h. The fabricated composites are denoted GO/P-BN/epoxy.

## 3. Results and Discussion

**Figure 1:**
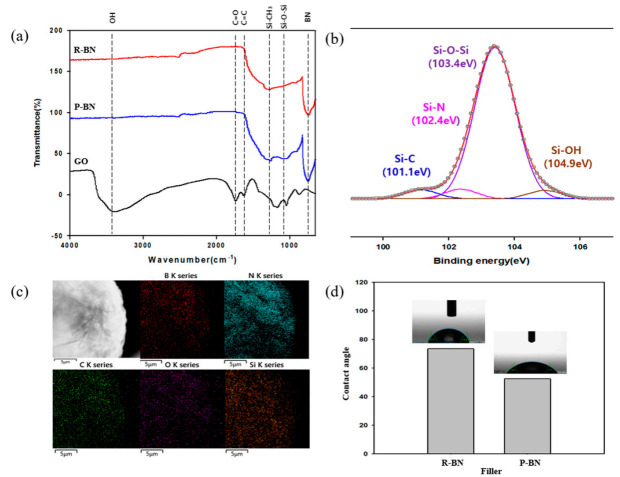
Confirmation of filler surface modification. (**a**) FT-IR spectra of fillers, (**b**) XPS Si2p deconvolution data of P-BN, (**c**) TEM-EDS image of P-BN, (**d**) ETDS contact angle of fillers.

The IR spectra of the R-BN and P-BN in Figure 1a both display the typical peaks of oxygen-containing groups, including those of OH at ~3400 cm^−1^, the C=O stretching at 1750 cm^−1^, and the sp^2^-hybridized carbon group stretching at 1620 cm^−1^ [18]. In addition, the peaks at 820 cm^−1^ (out-of-plane B–N–B bending vibration) and 1360 cm^−1^ (in-plane B–N stretching vibration) are intrinsic to the BN [19]. Compared to the R-BN, the spectrum of the P-BN displays additional peaks due to the Si–CH_3_ (1250 cm^−1^), and Si–O–Si (~1030 cm^−1^) derived from the PSZ coating and thermolysis [20].

The XPS analysis of the P-BN is shown in Figure 1b. The peak-fitting performed using the Person7 peak shape software package reveals one large peak and three minor peaks. The primary peak at 103.4 eV is attributed to the Si–O–Si bonds, thus demonstrating the successful ceramization of PSZ by calcination at 1000 °C. The two minor peaks at 101.1 and 102.4 eV are attributed to the Si–C and Si–N, respectively, which comprise the main chain of the raw PSZ. The remaining minor peak at 104.9 eV represents the Si–OH that was formed by hydrolysis of the raw PSZ [16,21]. A comparison of the peak intensities indicates that the coating material consists primarily of Si–O–Si (siloxane) bonds, which are also present in the ETDS.

To investigate the elemental composition of the P-BN, EDS analysis was performed, as shown in Figure 1c. As expected, the elements B, N, Si, C, and O were successfully detected. Moreover, the EDS mapping images demonstrate the uniform distributions of these five atoms.

To investigate the improvement in affinity between the matrix and the filler due to surface modification, the ETDS contact angle was measured. Here, the R-BN and P-BN were each compressed into pellet form via the hot press method; then, ETDS (10 μL) was dropped onto the surface of each pellet. The contact angles between the ETDS and the R-BN and P-BN were 73.6° and 52.6°, respectively. Since both the P-BN and ETDS contain siloxane groups, the improved interfacial adhesion between the filler and the matrix is attributed to their structural similarity [10]. 

**Figure 2:**
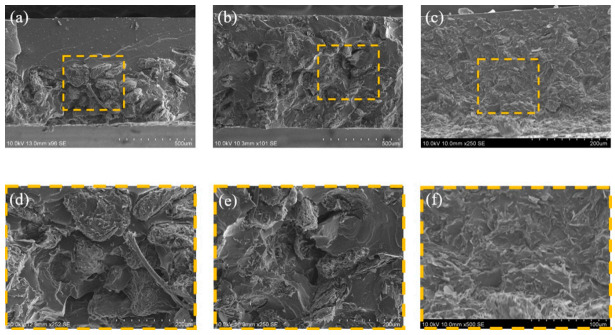
SEM cross section image of fabricated composites. (**a**) R-BN/epoxy composite, (**b**) GO/R-BN/epoxy composite, (**c**) GO/P-BN/epoxy composite, (**d**) magnified area of (**a**) image, (**e**) magnified area of (**b**) image, (**f**) magnified area of (**c**) image.

The morphologies of the epoxy composites were investigated by cross-sectional SEM microscopy. Thus, the SEM image in Figure 2a reveals a distinctly layered morphology due to the tendency for the high-density R-BN to diffuse downwards during the curing process. This phenomenon impairs the dispersion of fillers in the matrix and adversely affects the performance of the composites. The interfacial adhesion between the filler and the matrix was also revealed by the SEM images, with numerous gaps being observed around the filler due to the poor wettability of the R-BN (Figure 2d). These gaps may obstruct the flow of heat and result in heat loss. However, the introduction of GO into the composite is seen to improve the level of dispersion of the filler in the matrix (Figure 2b). This may be attributed to several possible reasons. First, an increase in the viscosity of the matrix is advantageous for reducing the mobility of BN during the mechanical mixing and curing processes, which has previously been shown to sink in the bottom of the matrix [22]. It is well known that the addition of GO increases the viscosity of the matrix due to the high aspect ratio of GO. Secondly, steric hindrance between two types of fillers in a composite material has been shown to affect the behavior of the filler [23]. Thus, the well-dispersed GO also helps prevent the BN from diffusing downwards and improve the level of BN dispersion in the matrix. However, an examination of Figure 2e indicates that the interfacial adhesion was not greatly improved. This residual problem was addressed by the successful surface modification of the BN to provide structural similarity with the ETDS matrix, as shown in Figure 2f. Thus, after calcination at 1000 °C, both PSZ and ETDS contained siloxane bonds in the main chain. As previously reported, structural change on the surface of BN increases the compatibility between the filler and the matrix [10].

**Figure 3:**
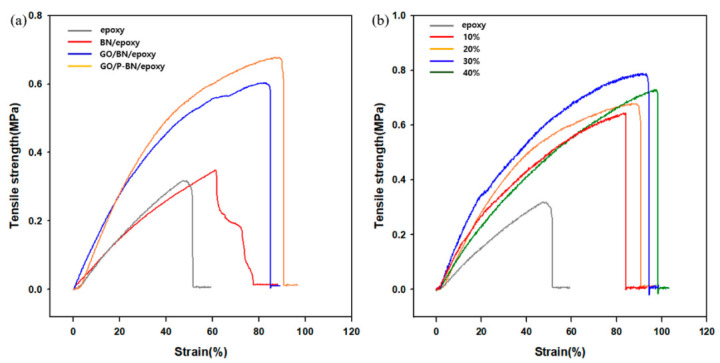
Mechanical property measured by UTM. (**a**) tensile strength measured for confirming the effect of surface modification and GO incorporation; (**b**) tensile strength measured for confirming the effect of P-BN weight fraction in a GO/P-BN/epoxy composite.

The tensile strengths of the various samples were measured to examine the effects of GO introduction and surface modification with PSZ, and the results are presented in Figure 3. The tensile strength of the neat epoxy is seen to be 0.31 MPa, whereas that obtained after the incorporation of GO is quite different (0.6 MPa). This can be explained by the morphology of the composite shown in the SEM cross section, where the R-BN was seen to aggregate at the bottom of the matrix, thus leading to a variation in the composition of the sample depending on the position. Hence, while the upper part of the matrix is compositionally similar to the neat epoxy, a much higher concentration of R-BN is present at the bottom of the matrix. Due to this drastic variation in the composition of the composite, the sample was broken twice during the tensile test. At the first break, the tensile strength was similar to that of the neat epoxy, while a decrease in tensile strength was observed at the second break. However, after GO introduction, the tensile strength of the BN/GO/epoxy composite was improved by 93% compared to that of the neat epoxy due to the enhanced dispersion of the filler in the presence of GO. Thus, the tensile strength of the composite is determined by the level of dispersion of the filler along the applied load direction [24]. When the filler is well dispersed in the matrix, the area over which load can be transferred is increased compared to that in the R-BN/epoxy composite. As the load is applied to the composite, cracks and voids therein begin to propagate and, when a crack approaches the filler, the stress dramatically increases in intensity until filler-matrix debonding occurs. In the case of the P-BN/GO/epoxy composite, the interfacial adhesion between the filler and the matrix was improved, as shown in the SEM cross-sectional image, so that the filler-matrix debonding was less likely after surface modification. This resulted in a 13% increase in the tensile strength due specifically to the PSZ coating. Moreover, this improvement in tensile strength is seen to increase with increasing filler concentration up to 30 wt %, although a decrease in the tensile strength is observed at a filler concentration of 40 wt %. This can be attributed to the interruption of the crosslinking between the epoxy and the matrix in the presence of excess filler.

**Figure 4:**
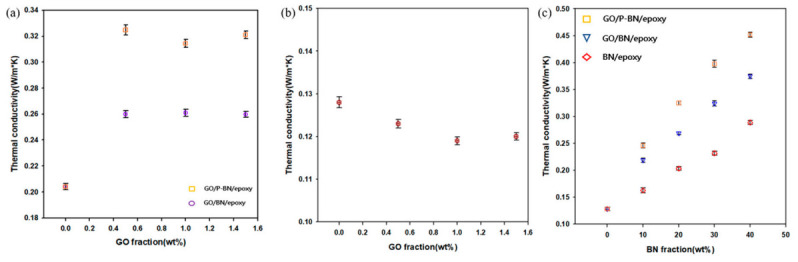
Thermal conductivity data of various samples. (**a**) thermal conductivity of GO/R-BN/epoxy composite depending on the GO weight fraction; (**b**) thermal conductivity of GO/epoxy composite depending on the GO weight fraction; (**c**) thermal conductivity of fabricated composites in this research.

The fact that intrinsic thermal conductivity of GO is very low (0.5–1.0 W·m^−1^·K^−1^) at room temperature was already well known in prior literature [25], which was also confirmed from Figure 4b. Similar thermal conductivities were achieved for composites incorporating GO fractions greater than 0.5 wt % as shown in Figure 4a. Based on these data, the optimum ratio of BN to GO was found to be 40:1 for both the GO/R-BN/epoxy and GO/P-BN/epoxy composites. With further increase in the BN fraction, the weight of GO incorporation also increased, but the thermal conductivity was not affected, as shown in Figure 4b.

**Figure 5:**
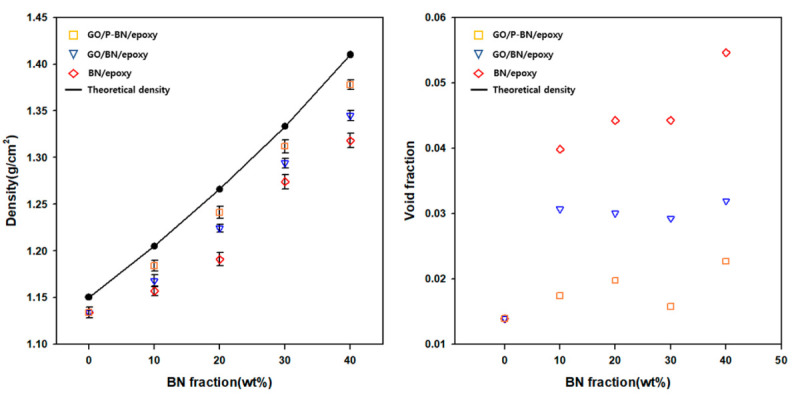
(**a**) theoretical expected densities and experimental densities; (**b**) void fraction of fabricated composites in this research.

To investigate the effects of surface modification and GO incorporation, the thermal conductivities of the composites were calculated and the results are presented in Figure 4c. In BN/ETDS composites, BN only existed at the bottom of the matrix, which cannot form a heat transfer path. As filler fraction increased, the filler occupied more area in the matrix and increased thermal conductivity. Furthermore, with the incorporation of GO, the BN is no longer aggregated at the bottom of the matrix, but, rather, becomes well dispersed throughout, so that the thermal conductivity of the GO/BN/epoxy composite is increased compared to that of the BN/epoxy composite. Moreover, the effect of surface modification is observed in the GO/P-BN/epoxy composite as the enhanced affinity between the filler and the matrix readily fill up any voids. To investigate the effect of the interface on the thermal conductivity, the void fractions of the composites were calculated from the theoretical and measured densities in accordance with the following equations:

void fraction = (ρ*_theoretical_* − ρ*_experimental_*)/ρ*_theoretical_*; (1)

and ρ*_theoretical_* = *1*/[(*W_filler_/ρ_filler_*) + (*W_matrix_*/ρ*_matrix_*)], (2)

where *W_filler_* and *W_matrix_* are the weight fractions of the filler and the epoxy matrix, respectively, and ρ*_filler_*, and ρ*_matrix_* are the densities of the filler and the epoxy matrix, respectively. It has previously been shown that much heat loss occurs at the voids in such composite materials due to the low thermal conductivity therein (thermal conductivity of air = 5 × 10^−5^) [26]. As shown in Figure 5, the GO incorporation and surface modification of BN both act to decrease the void fraction by improving the dispersion of the filler throughout the matrix.

**Figure 6:**
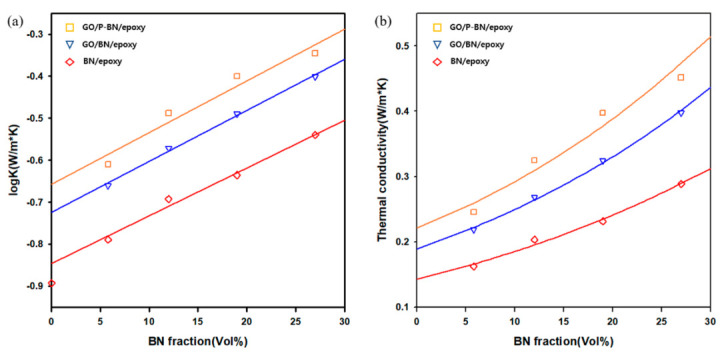
(**a**) logarithm of thermal conductivity of composites; (**b**) comparison of measured thermal conductivities of composites with calculated results using the Agari–Uno model.

The thermal conductivities of the composites were estimated using the Agari–Uno model, which is given by the following equation [27]:

log *K*_c_ = [(*V*_f_*C*_2_) × log *K*_f_] + [(1 − *V*_f_) × log(*C*_1_*K*_m_)] (3)

where *V*_f_ is volume fraction of fillers, *K*_m_ is the thermal conductivity of matrix, and *K*_c_ is the thermal conductivity of composites. Thus, *C*_1_ and *C*_2_ could be calculated from the slope and *y*-axis intercept, respectively, in Figure 6a. The values of *C*_1_ and *C*_2_ are generally between 0 and 1, with a *C*_1_ value close to 1 indicating that the filler is a suitable match for the polymer structure, and a *C*_2_ value close to 1 indicating that the thermally conductive route is well formed [28]. In the present work, the values of *C*_1_ and *C*_2_ were found to increase after the GO incorporation and surface modification, thus demonstrating that these factors are effective for improving the thermal conductivity in agreement with previous work [29].

**Figure 7:**
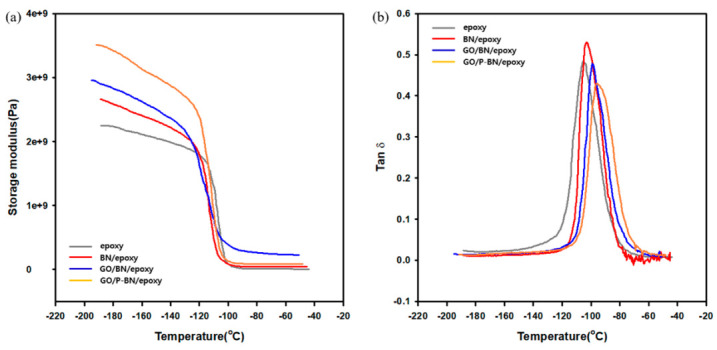
Mechanical property measured by DMA. (**a**) storage modulus of composites; (**b**) tan δ of composites.

The storage modulus provides information on the degree of elasticity and the load bearing ability of the composites [30] along with the stiffness, degree of crosslinking, and interfacial adhesion between the filler and the matrix [31]. The storage modulus of composites was measured by using the DMA graph as a function of temperature. In the present work, the storage modulus of the neat epoxy was found to be 2.2 GPa. In addition, it was continuously improved as methods in this research were applied. Thus, the efficient placement of the filler and the enhanced interfacial adhesion both increased the storage modulus as well as the thermal conductivity. However, the storage modulus was found to decrease as the temperature increased because the state of the polymer chains changed from the glass state to the rubbery state [32]. The glass transition temperature (*T*_g_) values were obtained from Figure 7b. The tan δ values display a similar tendency to the storage modulus. Overall, the dispersion of filler due to the incorporation of GO, along with the enhanced interfacial adhesion, resulted in a decreased mobility of the polymer chains and, hence, an increased glass transition temperature.

## 4. Conclusions

In the present study, dispersive and surface modification approaches were developed in order to improve the thermal conductivity of composites. The addition of GO was shown to increase the viscosity of the epoxy matrix by providing steric bulk, thus enhancing the filler dispersion. In addition, PSZ coating and thermolysis were shown to increase the structural similarity between the surface of the filler and the epoxy matrix. Due to these effects, the thermal conductivity of the GO/P-BN/epoxy was improved by 253% compared to that of the neat epoxy. Thus, it has been established that the effect of GO incorporation is to enhance the dispersion of the filler in the matrix and, thus, enhance the performance of the polymer composite.
